# Genome Sequence of Gammaproteobacterial *Pseudohaliea rubra* Type Strain DSM 19751, Isolated from Coastal Seawater of the Mediterranean Sea

**DOI:** 10.1128/genomeA.01208-14

**Published:** 2014-11-20

**Authors:** Stefan Spring, Anne Fiebig, Thomas Riedel, Markus Göker, Hans-Peter Klenk

**Affiliations:** Leibniz Institute DSMZ-German Collection of Microorganisms and Cell Cultures, Braunschweig, Germany

## Abstract

*Pseudohaliea rubra* strain DSM 19751^T^ is an aerobic marine gammaproteobacterium that was isolated from surface coastal seawater of the Mediterranean Sea. Here, we present its genome sequence and annotation. Genome analysis revealed the presence of genes involved in the synthesis of bacteriochlorophyll-*a* and the reserve compound glycogen.

## GENOME ANNOUNCEMENT

The gammaproteobacterial strain *Haliea rubra* CM41_15a^T^ (= DSM 19751^T^ = CIP 109758^T^ = MOLA 104^T^) was isolated from surface coastal water (depth, 3 m) of the Mediterranean Sea in the bay of Banyuls-sur-Mer, France (42°29′N 3°08′E) (1) and later reclassified as *Pseudohaliea rubra* (2). Its 16S rRNA gene sequence obtained by whole-genome sequencing is 97.5% identical to that of *Congregibacter litoralis* KT71^T^ and 95.4% identical to that of *Haliea salexigens* DSM 19537^T^. *P. rubra* belongs to the NOR5-3 lineage of the OM60/NOR5 clade, comprising aerobic anoxygenic photoheterotrophic bacteria commonly found in marine environments (2).

Strain *P. rubra* DSM 19751^T^ was selected for genome sequencing because of the expression of a light-harvesting complex with unusual spectral characteristics and its close phylogenetic relationship to the nonphototrophic species *H. salexigens* (3). A culture of DSM 19751^T^ was grown aerobically in DSMZ medium 514 (http://www.dsmz.de/catalogues/catalogue-microorganisms/culture-technology.html) at 28°C, and genomic DNA was extracted using the Jetflex genomic DNA purification kit (catalog no. 600100; Genomed), according to the manufacturer’s instructions, together with in-house modifications (4). DNA is available from the DSMZ through the DNA Bank Network (5). For whole-genome sequencing, a short paired-end library was sequenced using an Illumina MiSeq system. Library preparation was performed using the TruSeq DNA PCR-free sample preparation kit (Illumina, San Diego, CA), according to the manufacturer’s instructions, and yielded a mean insert size of 450 bp. The sequencing run resulted in 40,359,103 reads. To correct sequencing errors and improve the quality of reads, clipping was performed using fastq-mcf (http://code.google.com/p/ea-utils) and Quake (6). A total of 7,153,338 reads with a minimum length of 100 bp were assembled using Velvet version 1.0.18 (7). The gaps were closed using GapFiller version 1.10 (8) and manual editing in the Phred/Phrap/Consed package version 20.0 (9). The final assembly consists of 90 contigs, with an average coverage of 122.8. For automatic annotation and comparative genome analyses, the RAST annotation server was used (10). The determined draft genome sequence of *P. rubra* DSM 19751^T^ consists of 86 scaffolds, is 3.21 Mb in size, contains 2,860 protein-coding sequences, 3 rRNAs, and 41 tRNAs, and has a G+C content of 66.1%.

Genome analysis revealed the presence of a complete set of genes involved in photoheterotrophic growth based on bacteriochlorophyll-*a*. The light-harvesting complex of *P. rubra* has unusual spectral characteristics, displaying peaks at 804 and 821 nm, which indicates the expression of a peripheral LH3 complex. Interestingly, the protein sequence of the alpha subunit of the antenna complex of *P. rubra* (HRUBRA_02397) is more similar to the alphaproteobacterial *pucA*_c_ gene product of *Rhodopseudomonas palustris* strain TIE-1 (70% identity) than to the protein of the closely related species *C. litoralis* (57% identity), thereby indicating an adaptation of the antenna complex of both gammaproteobacteria to different ecological niches. In addition, several key metabolic features, including glycogen synthesis, distinguish *P. rubra* DSM 19751^T^ from *C. litoralis* KT71^T^ (11).

### Nucleotide sequence accession numbers.

The whole-genome shotgun project has been deposited at DDBJ/EMBL/GenBank under the accession no. AUVB00000000. The version described in this paper is version AUVB01000000.
